# A Meta-Analysis on the Antimicrobial Effectiveness of Ozonated Water Treatments for Fresh Produce Washing—Effect of Ozonation Methods

**DOI:** 10.3390/foods13233906

**Published:** 2024-12-03

**Authors:** Haknyeong Hong, Marissa Faye Rizzi, Danhui Wang, Lynne McLandsborough, Jiakai Lu

**Affiliations:** 1 Department of Food Science, University of Massachusetts, Amherst, MA 01002, USA; haknyeonghon@umass.edu (H.H.); lm@foodsci.umass.edu (L.M.); 2 Department of Nutrition and Food Sciences, Texas Woman’s University, Denton, TX 76204, USA

**Keywords:** meta-analysis, ozonated water, meta-regression, antimicrobial effectiveness, washing process, fresh produce

## Abstract

Due to the lack of a pathogen-killing process, foodborne outbreaks from contaminated fresh produce have been increasing worldwide. Hence, it is increasingly recognized that the washing step with sanitizers is important to control microbial contamination. Ozonated water is suggested as a substitute for chlorine-based sanitizers, addressing concerns about the effectiveness and environmental impact of chlorine-based sanitizers. However, using ozone as a sanitizer in the fresh produce washing process is still challenging because of its unstable and inconsistent antimicrobial effectiveness under various testing conditions. A meta-analysis was focused on the comparison of antimicrobial effectiveness between different ozonation methods commonly adopted in laboratory settings, including stationary pre-ozonated water, agitated pre-ozonated water, and sparging. The meta-analysis showed that the sparging method results in the highest microbial log reduction compared to other methods. We further developed meta-regression models based on three ozonation methods to identify key processing variables influencing the antimicrobial effectiveness of ozonated water. Attempts were made to link key processing variables to ozone stability and the mass transport phenomena involved in the washing process. This research will contribute to designing and developing a washing process to increase fresh produce safety by identifying key factors in each ozonation method and facilitate interlaboratory comparison studies.

## 1. Introduction

With growing interest in healthy and natural foods, there has been an increasing demand for minimally processed fresh produce (fresh fruits and vegetables) that preserves their essential nutrients and bioactive compounds from post-harvest processing [1]. As a tradeoff, there are no post-harvest processes for minimally processed fresh produce that sufficiently eliminate pathogenic microorganisms. Importantly, minimally processed fresh produce is usually consumed raw, which increases the risks of foodborne outbreaks due to contaminations of pathogenic bacteria, such as *Escherichia coli* O157:H7, *Salmonella* spp., *Listeria monocytogenes*, and so on [2,3].

To curb the potential risks, chlorine-based sanitizers have been conventionally used in post-harvest washing processes due to their low cost and acceptable level of microbial reduction [4,5]. While chlorine-based sanitizers are widely used, several studies have reported that they provide limited microbial reduction, achieving less than a 2-log reduction [6,7,8]. This has raised concerns about their effectiveness in reducing foodborne illness risks associated with fresh produce. More importantly, chlorine-based sanitizers are recognized for their potential to form carcinogenic by-products and their negative environmental impact [9,10].

With concerns centered around the effectiveness, safety, and environmental impact of chlorine as a sanitizer, alternative sanitizers and antimicrobial technologies for the fresh produce washing process have been investigated, such as peroxyacetic acid treatments, ozonated water treatments, electrolyzed water, ultraviolet radiation, ultrasound, and their combinations [11,12,13,14,15,16,17]. Ozonated water has emerged as a promising sanitizer for the fresh produce washing process [18,19]. Recent research highlights numerous advantages of using ozonated water as a sanitizer [20]. Firstly, ozone demonstrates a superior oxidizing power, attributed to its higher redox potential in comparison to chlorine, leading to a higher microbial reduction [21,22]. Secondly, ozone exhibits a strong antimicrobial ability against a wide range of microorganisms due to the generation of a non-selective reactive oxygen species as active antimicrobial agents [23]. Thirdly, ozone has been considered a Generally Recognized As Safe (GRAS) antimicrobial agent in food production processes by the United States Food and Drug Administration [24]. Last but not least, ozone is eco-friendly as it decomposes into oxygen in liquid form, leaving less harmful residual components during the process in most of the processing conditions [25].

The antimicrobial effectiveness of ozonated water for fresh produce washing has been extensively studied in the laboratory settings under a wide range of process conditions, including ozone concentration, treatment time, temperature, pH, ozonation methods, fresh produce types, types of foodborne pathogens, and more [26,27]. However, it is important to note that the reported antimicrobial effectiveness of ozonated water varies significantly across different studies, and, in some cases, conflicting results have been observed [26,28]. For instance, studies have shown that a higher initial ozone concentration and longer treatment time contribute to greater microbial reductions [29,30,31]. On the other hand, other studies have indicated that a longer treatment time does not significantly impact the antimicrobial effectiveness of ozonated water [32,33]. Additionally, the effect of the temperature on the antimicrobial effectiveness of ozonated water remains elusive, partly because temperature influences ozone stability and its reaction rate in opposite ways [34,35]. The varying antimicrobial effectiveness reported in the current literature arises from the unstable nature of ozone. This instability is heavily influenced by specific process conditions, making it challenging to predict and control the effectiveness of ozonated water as an antimicrobial agent [36].

To the best of our knowledge, there has been no systematic evaluation of the ozonated water efficacy in reducing foodborne pathogens on fresh produce. As an initial step, we conducted a meta-analysis to quantitatively evaluate the antimicrobial effectiveness of ozonated water on fresh produce. The substantial range of antimicrobial effectiveness noted in the meta-analysis emphasizes the importance of comprehensively understanding the intricate nature of ozone and its susceptibility to interference from process conditions when utilized as a sanitizer in the fresh produce washing process. To explore this, we further developed meta-regression models focusing on three primary ozonation methods commonly adopted in laboratory settings: stationary pre-ozonated water, agitated pre-ozonated water, and sparging. These models aim to identify critical process variables of the antimicrobial effectiveness of ozonated water across these different ozonation methods, providing a guideline for optimizing its application in fresh produce washing.

## 2. Materials and Methods

### 2.1. Data Collection

The protocols and guidelines for meta-analysis were conducted by following the PICO (population, intervention, control, and outcome) process [37]. The first factor, “population”, refers to fresh produce types—fruits and vegetables in this case. Fresh produce is defined as raw fruits and vegetables that are typically consumed in their fresh state without cooking or processing. The second factor, “intervention”, specifies the ozonated water treatments. The third and fourth factors, “control” and “outcome”, are characterized by the survival population of the pathogen before and after the treatment on fresh produce surfaces, respectively. The pathogens referenced in this review paper were based on the frequency of outbreaks related to fresh produce. The pathogens included *E. coli* O157:H7, *E. coli* O157:H7 surrogate, *Salmonella* spp., *Listeria monocytogenes*, and *L. monocytogenes* surrogates, such as *Listeria innocua*.

The literature identification was conducted in April 2023 using multiple academic search engines, including Google Scholar, PubMed, and Web of Science. To collect relevant data for meta-analysis, the search employed the following keyword combinations: “Aqueous ozone” or “Ozonated water” or “Ozone solution” AND “*Escherichia coli* (*E. coli*)”, “*Salmonella*”, “*Listeria monocytogenes* (*L. monocytogenes*)”, or “pathogen(s)” or “foodborne pathogen(s)” AND “fresh produce” or “vegetables” or “fruits”, AND “control” or “inactivation” or “reduction” or “sanitation” or “disinfectant” or “washing”. Data included studies available in scientific journals from 1960 to April 2023.

### 2.2. Inclusion Criteria, Evaluation of Study Quality, and Data Extraction

**Figure 1:**
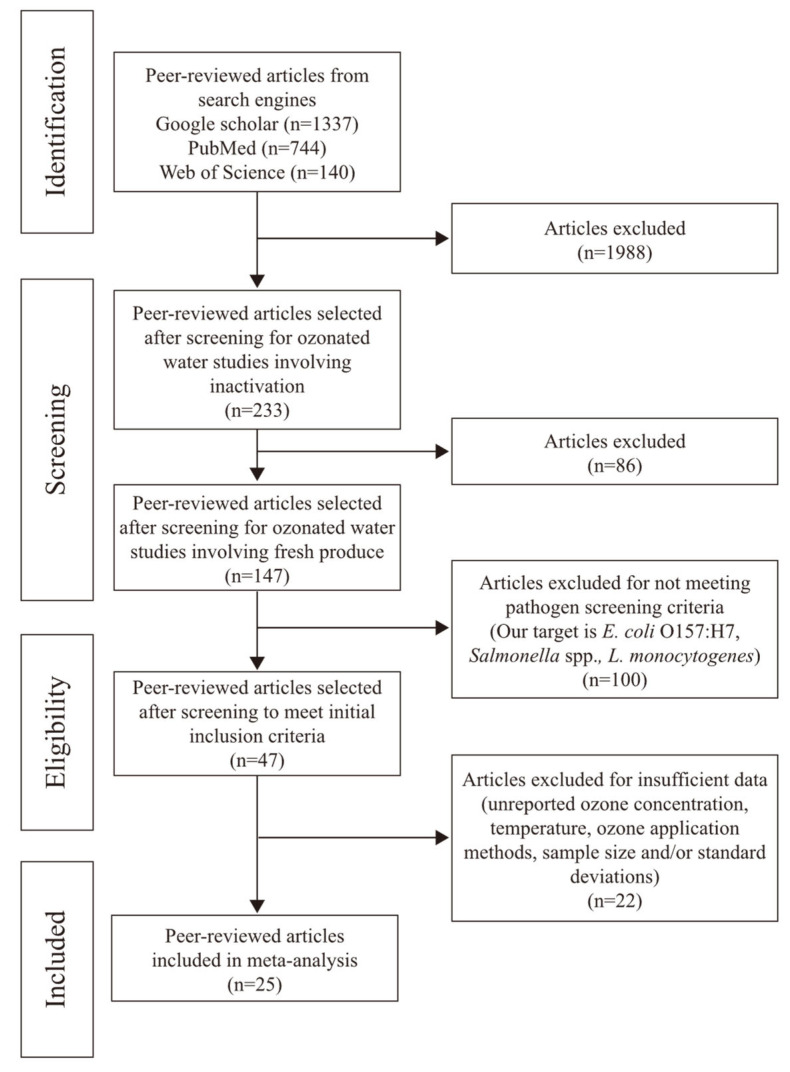
Flow diagram showing the systematic review process and the selection of studies for inclusion in the meta-analysis.

As shown in Figure 1, these studies were screened by the steps outlined in Moher et al. (2010) [37]. To begin with, in the identification step, thesis and review papers were eliminated to prevent duplicate data. Only studies related to microbial inactivation on fresh produce were included in the primary data; studies evaluating other aspects, such as fresh produce quality and shelf life, were rejected. Studies using treatment methods such as cold plasma or gaseous ozone treatments were also excluded. In the screening step, studies that used other food types, food contact surfaces, cross-contamination, or wastewater were rejected. Only studies related to the inactivation of fresh produce were included. After that, as a third criterion, the primary study had to clearly report the pathogens (*E. coli* O157:H7, *E. coli* O157:H7 surrogate, *Salmonella* spp., *L. monocytogenes*, and *L. monocytogenes* surrogate), not other pathogenic bacteria such as *Vibrio parahaemolyticus*, *Bacillus cereus* or a broad range of bacteria, viruses, and fungi. As a final criterion, papers were eliminated if they did not specify the following standard: ozone concentration, ozone generation method, the temperature of the treatment solution, the population of pathogens both before and after treatment, or the log reduction in a pathogen, sample size, and/or standard deviations. Considering all these requirements, 25 primary studies were included in this meta-analysis.

### 2.3. Description of Primary Studies and Data for Meta-Analysis

Table 1 summarizes 25 primary studies, including 217 individual observations and supplements further details on study characteristics categorized by ozonation methods. Following the preliminary search, the literature on ozonated water highlighted seven predominant factors that were expected to influence its antimicrobial effectiveness [9,21]. However, it is essential to emphasize that the data regarding study characteristics did not encompass a crucial factor: pH. This omission occurred because most primary studies did not provide pH values for their process conditions [38]. Consequently, six variables were considered as study characteristics in order to elucidate the variations in the antimicrobial effectiveness of ozone: ozone concentration, treatment time, ozonated water temperature, inoculated microorganisms, fresh produce types, and inoculation methods.

Ozone concentration data collected from primary studies represented initial dissolved ozone concentration or residual ozone in the solution after treatment. The changes in concentration range from 0.15 to 36 mg/L. The treatment time ranges from 0.5 to 120 min. The temperatures of ozonated water range from 4 to 50 °C. The inoculated microorganisms consisted of *E. coli* O157:H7, *E. coli* O157:H7 surrogate, *Salmonella* spp., *L. monocytogenes*, and *L. monocytogenes* surrogate. The attachment time of the microorganisms on fresh produce was not detailed. Inoculation procedures were therefore partitioned into three groups based on the inoculation method: (1) Dipping inoculation: samples are immersed in a bacterial culture solution with or without agitation and then dried for a few hours. (2) Dipping and incubation: samples are submerged into a bacterial culture solution and then incubated at a specific temperature and time. (3) Spot inoculation: drops of the bacterial inoculum are deposited on the surface of fresh produce and then dried for a certain time.

### 2.4. Parameterization of Effect Size

In order to compare the data collected from 25 different studies, microbial mean log reductions were selected as the effect size. The data collected from primary studies were expressed as a continuous variable (survival population of pathogens in fresh produce before and after treatment). The possible parameters to measure effect size are the raw (unstandardized) mean difference, standardized mean difference, and response ratios [60]. Among these methods, the raw mean difference was most suitable to apply in this meta-analysis to measure the effect size because all the data collected from primary studies were reported in the same log CFU scale to show the difference between the control and treatment means. Moreover, this unit is a meaningful parameter because it intuitively provides the number of inactivated bacteria and has been widely used among microbiologists and the food industry.

However, some studies reported the degree of inactivating pathogens as a log reduction, while others described the survival population of pathogens before and after treatment. If studies reported the log reduction in pathogens with standard deviations, then, the log reduction was used to express the effect size. Alternatively, if studies reported the survival population of a pathogen before and after treatment, then, the subtraction of the population after treatment from the initial population was used to indicate the effect size. Therefore, the primary study, j, reports the mean for two groups (initial population or control or pre- and post-populations). X_C_ and X_T_ are the means of the initial population (control) and post-populations, respectively. The effect size (MLR) is defined as:

(1) $${MLR}_{j}=X_{Cj}-X_{Tj}$$

S_C_ and S_T_ represent the standard deviation of each group, and n_C_ and n_T_ are the sample sizes used in each group, before and after treatment, respectively. The standard errors (SE) of the mean log reduction (MLR) can be estimated as:

(2) $$SE\left({MLR}_{j}\right)=\sqrt{\left(\frac{S_{Cj}^{2}}{n_{Cj}}+\frac{S_{Tj}^{2}}{n_{Tj}}\right)}$$

The sparseness of the data had some implications in the choice and the design of the meta-analysis mixed-effect models [61]. The meta-analysis was conducted in the R (version 4.3.2) programming environment using the “metafor” package (version 4.4-0) [62].

### 2.5. Meta-Regression Model

Meta-regressions are multi-variable analysis tools that use individual study characteristics as independent variables and outcomes of primary studies as dependent variables. We developed individual meta-regression models based on the ozonation methods and the crucial process conditions that impact the antimicrobial effectiveness of ozonated water across these different ozonation methods. Meta-regression models were composed of the same moderators containing continuous and categorical variables, as detailed in Table 2. The continuous variables in this study were ozone concentration and treatment time. The categorical variables in this study were ozonated water temperature, inoculated microorganisms, fresh produce types, and inoculation methods. Meta-regression models were run for microbial mean log reductions in the R software version 4.3.2. A mixed-effects model was used to describe the microbial mean log reduction (MLR).

The MLR is modeled as:

(3) $$MLR_{n}=\beta_{0}+\beta_{1n}\;Ozone\;concentration+\beta_{2n}\;Treatment\;time+\beta_{3n}\;Temperature+\beta_{4n}\;Inoculated\;microorganisms+\beta_{5n}\;Fresh\;produce\;types+\beta_{6n}\;Inoculation\;method+\varepsilon_{n}$$

where β_o_ is the mixed-effects intercept for group n, β_1n_ is a vector representing the effects of the ozone concentration (mg/L), β_2n_ is a vector describing the effects of the treatment time (min), β_3n_ is the fixed effects of the ozonated water temperature, β_4n_ is the fixed effects of the inoculated microorganisms, β_5n_ is the fixed effects of the fresh produce type, β_6n_ is the fixed effects of the inoculation method, and ε_n_ is the residual error. The residual error was assumed to follow a normal distribution. The meta-regressions used baselines for any continuous variables and categorical variables as a default to calculate intercepts (Table 2).

#### 2.5.1. Description of Categorical Variables for Meta-Regression Model

We treated the temperature as a categorical variable and categorized the temperature ranges as low (below 15 °C), middle (15 to 25 °C), and high (above 25 °C) because the distribution of the available temperature used in all the studies was centered around 5, 22, and 40 °C. The fresh produce types considered in this study were treated as a categorical variable. However, it is important to note that the available data for fresh produce were limited and largely unbalanced, posing challenges in performing the meta-regressions. There was a scarcity of data for meta-analysis, with fewer data values available for certain types of fresh produce. For instance, microbial log reductions were only reported for *Salmonella* spp. in the case of green onion and for *E. coli* O157:H7 in the case of cabbage. Furthermore, there was only one available observation for microbial log reductions in *Salmonella* spp. on turnips. As a result, fresh produce types were grouped into three distinct categories based on their surface characteristics and unique geometry, as reported in the literature [63,64,65,66,67]:

1. Green leaves category, which includes lettuce, cabbage, spinach, parsley, green onion, red chard, basil, and bok choy.
2. Rough surface and complex geometry category, which comprise alfalfa sprouts, apple stem–calyx, carrots, mung bean sprouts, oyster mushrooms, raspberries, strawberries, and turnips.
3. Smooth surface category, which consists of apple surface, blueberries, and cherry tomato (or grape tomato).

#### 2.5.2. Description of Different Groups for Meta-Regression Model

The ozonation methods for fresh produce washing varied across different primary studies. However, there has been insufficient focus on investigating these ozonation methods as a crucial factor influencing antimicrobial effectiveness during the washing process, especially when comparing experimental results across different laboratories reported in the literature. We hypothesize that the way ozonated water is applied can directly influence both the stability of ozone and mass transport phenomena involved in the transfer and interaction between the active compounds, including dissolved ozone and reactive oxygen species and targeted microorganisms.

**Figure 2:**
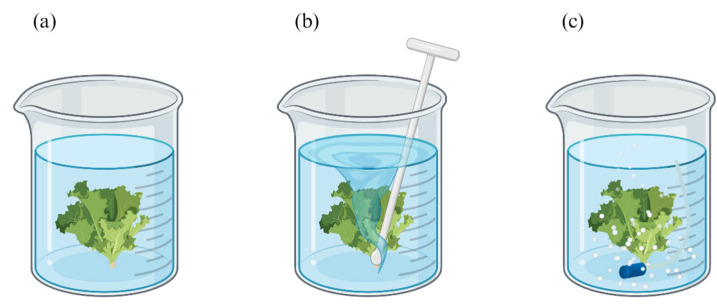
Three ozonation methods for fresh produce, including (**a**) stationary pre-ozonated water, (**b**) agitated pre-ozonated water, and (**c**) sparging.

Consequently, different ozonation methods can significantly impact antimicrobial effectiveness. In this paper, we focused on the most prevalent lab-scale methods, which we categorized into three main ozonation methods (Figure 2). These methods were categorized based on their effects on both ozone stability and mass transport characteristics related to the washing process. We analyzed the overall size effect under each method and developed separate meta-regression models to identify relevant process conditions under different ozonation methods. The three ozonation methods for fresh produce washing are as follows:

1. Stationary pre-ozonated water method: Samples are placed in pre-ozonated water without agitation. In these cases, ozone degradation primarily depends on the temperature, pH, and organic loads. The effectiveness of antimicrobial activity is mainly limited by the diffusion rate of the active antimicrobial compounds, considering the fast reaction rate of the antimicrobial process of ozonated water [36].
2. Agitated pre-ozonated water method: Samples are immersed in pre-ozonated water within a container and agitated using tools such as a magnetic stirrer, shaker, manual stirring, or a glass rod. This method significantly improves the distribution of the active antimicrobial compounds throughout the solution and increases their contact with targeted microorganisms on the produce surfaces. However, increased agitation leads to faster ozone degradation due to higher turbulence levels.
3. Sparging method: Ozone gas is introduced into water by bubbling it with or without agitation, using a sparger with fixed pore sizes. This method not only enhances the mass transfer rate of ozone and effectiveness of active antimicrobial compounds against microorganisms but also maintains a high level of reactive oxygen species as active ingredients during the process.

Several industrial flume washing processes use a continuous ozonation system via a side stream to maintain a constant dissolved ozone concentration during washing. However, this method has been rarely studied in laboratory settings. Due to the limited number of studies [68] meeting our inclusion criteria, we did not include this type of ozonation application method in our study.

## 3. Results and Discussion

### 3.1. Meta-Analysis of the Effect Size

**Figure 3:**
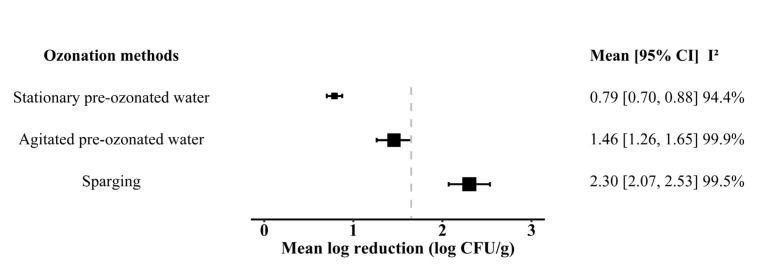
Forest plots showed the effect size of different groups classified by ozonation methods. The dashed line represents the overall estimated effect size (1.65 log reduction) of the intervention.

When all observations from primary studies were conducted for meta-analysis, the overall estimated effect size of the intervention was 1.65 ± 0.14 log reduction with a high level of heterogeneity, estimated in the form of I^2^ at 99.8%. It is well known that such a high level of heterogeneity can be explained by the differences found in study characteristics or population studies rather than random error [69]. Instead of pursuing a single effect size estimate, the main goal of this meta-analysis shifted towards investigating the causes of heterogeneity [70], which are the different process conditions of fresh produce washing in this paper. To explore the potential variations in the significance of process conditions, we hypothesized that different ozonation methods might yield differing outcomes. Consequently, we partitioned our data according to the ozonation methods before constructing the meta-regression models (Table 1). The meta-analysis of the estimated effect size also shows a significant difference between the three ozonation methods, as described in Figure 3. The sparging method has the highest estimated log reduction, with a summary effect at 2.30 log CFU/g, whilst the agitated pre-ozonated water and stationary pre-ozonated water methods have an estimated log reduction of 1.46 log CFU/g and 0.79 log CFU/g, respectively. Utilizing the sparging method for fresh produce washing significantly enhances the antimicrobial effectiveness of ozonated water, surpassing that of other methods. Importantly, the I^2^ remains high for the three ozonation methods (Figure 3), confirming the need to further explore the potential variations causing heterogeneity.

### 3.2. Meta-Regression Models

We developed meta-regression models to explore the effect of process conditions under different ozonation methods. In each of the three ozonation method models, the process conditions are included as moderators/predictors to evaluate their impact on the antimicrobial effectiveness of ozonated water. In this meta-regression model, we considered a moderator to be statistically significant when *p* < 0.05.

#### 3.2.1. Meta-Regression Model by Stationary Pre-Ozonated Water

The results presented by the stationary pre-ozonated water model are shown in Table 3. The moderators/variables included in the development of the models are ozone concentration, treatment time, ozonated water temperature, inoculated microorganisms on fresh produce, fresh produce types, and inoculation methods. The meta-regression model shows that the antimicrobial effectiveness of ozonated water is influenced by several significant moderators, including ozone concentration, temperature, fresh produce types, and inoculation methods.

The β_1n_ values show that the antimicrobial effectiveness of ozonated water was positively correlated to the initial ozone concentration. Higher initial dissolved ozone concentrations result in a larger microbial log reduction at a rate of 0.063 log CFU/g per 1 ppm increase in dissolved ozone concentration. Higher initial concentrations of dissolved ozone lead to increased levels of dissolved ozone and reactive oxygen species, and quicker transfer rates of both active antimicrobial compounds toward specific microorganisms. This enhanced mass transfer rate boosts the effectiveness of the antimicrobial action [18]. Based on the β_3n_ values, it is evident that, at higher temperature levels, ozonated water has larger antimicrobial effectiveness in the stationary pre-ozonated water method. While higher temperatures may lead to faster ozone decomposition [71], it is important to note that the hydroxyl radicals produced during this process are notably potent reactive oxygen species. These radicals react non-selectively with microorganisms and organic compounds [35]. This trend is consistent with the findings by Xu et al. (2014), indicating that higher heat treatments resulted in more inactivation of *Salmonella* spp. on tomatoes and lettuces than at room temperature [30]. β_5n_ parameters suggest that the antimicrobial effectiveness of ozonated water depends on the characteristics of the fresh produce treated with the stationary pre-ozonated water method. Among fresh produce types, microorganisms on produce with rough surfaces and complex geometries are significantly more difficult to inactivate than microorganisms on green leaves fresh produce types. These differences can be explained by the characteristics of the surface of fresh produce, where the rough surface can enhance microbial attachment while hindering microbial detachment and inactivation [72]. β_6n_ parameters provide further insights into the different microbial reductions achieved when different inoculation methods are used. Dipping and incubation, and spot inoculation methods show a higher microbial reduction than the dipping method. Previous research indicates that bacteria inoculated using dipping and incubation methods tend to be more challenging to control with sanitizers due to the increased attachment and aggregation of bacteria on the surface of fresh produce, increasing their adhesion strength [73]. Including only one observation in the determination of β_6n_ for the dipping and incubation inoculation may have introduced a potential bias to the results, suggesting that the dipping and incubation inoculation leads to a greater microbial log reduction. The dipping inoculation method may be more difficult to control since it allows the penetration of bacteria into the cut edges of fresh produce, making it more challenging for ozone to effectively reach and inactivate them [74]. However, it could be argued that this may be more representative of the conditions that may be encountered during the processing of fresh produce.

The β_2n_ parameter indicates that treatment time is not a significant factor. This may be explained by rapidly reaching a nonlethal threshold concentration of ozone because of a reaction in the water. It can also be explained by a slow diffusion rate of active antimicrobial compounds, including ozone molecules and reactive oxygen species toward microorganisms [36]. For example, the stationary pre-ozonated water treatment causes non-significant antimicrobial effectiveness on alfalfa sprouts, even with an increase in the treatment time from 2 to 64 min [32]. The β_4n_ values indicate that the antimicrobial effectiveness of ozonated water does not depend on the inoculated microorganisms. All three pathogens were similarly susceptible to ozonated water. This result is similar to the previous study about the susceptibility of Gram-positive and Gram-negative bacteria to ozone [36]. The antimicrobial effectiveness of ozonated water across bacteria can be attributed to the ozone’s non-selective inactivation of bacteria and the complexity of the two pathways, including direct and indirect reactions [36].

#### 3.2.2. Meta-Regression Model by Agitated Pre-Ozonated Water

The results for the meta-regression model by agitated pre-ozonated water method are presented in Table 3. The antimicrobial effectiveness of ozonated water in this model is attributed mainly to three moderators: treatment time, fresh produce types, and inoculation method. The β_2n_ values show increasing the treatment time enhances the antimicrobial effectiveness by 0.071 log CFU/g per min. When washing fresh produce under agitation, active antimicrobial agents like ozone molecules or reactive oxygen species are more likely to be uniformly distributed throughout the solution. This increases their chances of contacting and inactivating microorganisms on the fresh produce. Since the antimicrobial action of ozonated water is very fast [75], its effectiveness appears to be more limited by the probability of contact between microorganisms and the antimicrobial agents rather than their concentration. This is consistent with our findings, which show that treatment time is now more important than ozone concentration. The longer the exposure time, the higher the probability of contact, and therefore, the greater the antimicrobial effect. For example, more reductions in *E. coli* O157:H7 were observed after 10 min of ozonated treatment than after 1 or 5 min with the same ozone concentration [33]. However, it is important to note that microbial log reduction can be expected to increase with an increase in the treatment time when the ozone concentration is above the nonlethal threshold level. β_5n_ parameters indicate that, when using agitated pre-ozonated water, the antimicrobial effectiveness varies depending on the specific attributes of the fresh produce being treated. Microorganisms on smooth surfaces are significantly more susceptible to inactivation by ozonated water compared to those present on green leaves fresh produce types. Smooth surfaces may allow more direct contact between ozone and microorganisms, enhancing the antimicrobial effectiveness of ozone. In contrast, the fibrous structure of rough surfaces and the complex geometries of other produce types may provide more opportunities for pathogens to hide from direct ozone exposure, thereby reducing the effectiveness of the treatment. The β_6n_ values demonstrate that the spot inoculation method is vulnerable to ozonated water treatment compared to the dipping method. This enhanced susceptibility to the ozonated water treatment in the spot inoculation method may be attributed to the localized distribution of bacterial cells on the produce surface, potentially increasing their exposure to the sanitizer. In contrast, the dipping method potentially allows bacteria to penetrate deeper into the lettuce tissue, making them less accessible to the sanitizer [74]. Similar results observed between the dipping method and the dipping and incubation method can be attributed to the wide variation in incubation times (ranging from 2.5 h to 24 h) used in the latter. This finding is consistent with a study that reported no significant difference in the reduction in *E. coli* on lettuce treated with ozone after incubation between 4 °C for 18 h and 22 °C for 6 h because the development of cell aggregates increased after 18 h of incubation at 22 °C [57]. Therefore, it suggests the importance of specifying the appropriate time and temperature conditions required for bacteria to form aggregates, serving as a potential barrier to chemical sanitizers.

β_1n_ values indicate that the initial ozone concentration is not a significant moderator. We assumed that agitation facilitates ozone transfer to the targeted microorganisms and further deems substantial changes in the initial ozone concentration less important since the antimicrobial action of ozone is determined by ozone transfer. The temperature is not a significant moderator, possibly due to insufficient temperature data, particularly at high temperatures during the agitated pre-ozonated water method. This also may be attributed to the balance between ozone reactivity and ozone decomposition, which is influenced by the temperature. The β_5n_ values indicate that the antimicrobial effectiveness of ozonated water is reasonably consistent across different fresh produce types. This is because ozone transfer is the main factor determining the antimicrobial effectiveness in the agitated pre-ozonated water method. However, it is important to note that our categorization of fresh produce types did not systematically consider their surface characteristics, such as contact angle, wax composition, and hydrophobicity. The absence of this detailed information could potentially affect our results in this model, highlighting the importance of conducting further research in this area.

#### 3.2.3. Meta-Regression Model by Sparging Method

In the meta-regression model by the sparging method, the significant moderators are the treatment time, fresh produce types, and inoculation method, which contribute to the antimicrobial effectiveness of ozonated water. Notably, the intercept is statistically significant at approximately 1.094 log CFU/g, indicating that the sparging method is highly effective under most conditions, even when the concentration and treatment time are very low. This baseline effectiveness is well demonstrated by Ölmez et al. (2009), where the lettuce was treated with ozonated water with the sparging method [40]. They achieved approximately 2 log microbial reductions with 1.5 ppm of ozone at a 2-min treatment time. In contrast, the dipping method resulted in 0.8 log microbial reductions using a higher ozone concentration and longer treatment time [31]. The higher effectiveness of ozonated water in the sparging method can be attributed to the continuous supply of gaseous ozone by bubbles, which increase the contact area between water and ozone, promoting an efficient microbial reduction. The effectiveness may also be attributed to the additional physical interaction between bubbles and the contaminated surface [76,77]. The β_2n_ value indicates an estimated microbial reduction of 0.024 log CFU/g for every minute increase in the treatment time. Given the rapid antimicrobial action of ozonated water, its effectiveness in the sparging method, similar to the agitated pre-ozonated water method, seems to be more limited by the likelihood of contact between microorganisms and the antimicrobial compounds than by the concentration of ozone. Additionally, this can be explained by the fact that the sparging method significantly enhances the mass transfer rate of ozone and diffusion. The constant introduction of ozone gas maintains active antimicrobial compounds, including dissolved ozone and reactive oxygen species in water, surpassing the capabilities of the agitated pre-ozonated water method [78]. For example, bubbling ozone causes more log reductions in *E. coli* O157:H7 in apples (3.7 log CFU/g) than agitated pre-ozonated water (2.6 log CFU/g) [48]. Similarly, the efficacy of bubbling ozone has a higher impact on microbial reductions than that of the stationary pre-ozonated water used on lettuce [57,79]. The β_5n_ values demonstrate that rough surface and complex geometry groups are significantly more difficult to inactivate than green leaves. This trend aligns with the results observed in the stationary pre-ozonated water method model, suggesting that controlling bacteria on fresh produce types with rough surfaces or complex geometries is particularly challenging with ozonated water. As explained for the β_6n_ values, bacteria inoculated by the spot methods on fresh produce demonstrate a higher susceptibility to the ozonated water treatment compared to the dipping inoculation method. This finding is consistent with the results observed in the agitated pre-ozonated water method. We also assumed that the initial population of microorganisms may contribute to these results, given that the dipping inoculation method groups had relatively higher initial populations than other inoculation methods. Therefore, it highlights the specifying the initial population of microorganisms for a more accurate evaluation of the antimicrobial effectiveness of ozonated water.

The ozone concentration and temperature are not significant factors in this model. This suggests that the effectiveness of the sparging method is less influenced by the initial ozone concentration and temperature variations since the ozone reactivity is governed by the mass transfer rate of ozone and its continuous supply of gaseous ozone to water, which helps to maintain the dissolved ozone concentration at a constant level. The values of β_4n_ indicate that the antimicrobial effectiveness of ozonated water does not depend on inoculated microorganisms, which is consistent with the results found in other models. These results highlight the unique advantages of the sparging method in the ozonated water treatment, emphasizing its effectiveness even under low concentration and short treatment time conditions.

### 3.3. Possible Reasons for the Remaining Heterogeneity in Meta-Regression Models

Although the meta-regression models using the ozonation method can help to better understand the process conditions influencing microbial reduction, one should note that the I^2^ remains high for three ozonation methods (Table 3). This indicates that numerous factors, not yet fully explored in the current literature, could elucidate the disparities in the microbial log reduction, especially those directly influencing the physicochemical properties of ozone, such as stability, solubility, and reactivity.

First, the pH could be a factor that affects the antimicrobial effectiveness of ozonated water on fresh produce washing because there is a controversial result related to the effect of the pH level. Our final meta-regression model does not include a pH value as a moderator because of data sparseness. Previous studies indicate that ozonated water at a low pH level increases microbial reduction [20]. This is attributed to the increased stability of ozone in acidic solutions, where the influence of hydroxyl ions decreases [80,81]. For instance, ozonated water with citric acid (pH 2.64) on tomato and lettuce has a higher microbial log reduction than ozonated water [30]. Conversely, a higher microbial log reduction is achieved at higher pH levels under the same ozone concentration and treatment time [82]. The role of hydroxyl radicals can explain this difference at different pH levels.

Another contributing factor could be the detail of ozonation methods because the antimicrobial effectiveness of ozonated water can be improved with physical treatment or mechanical forces. There are different mechanical forces, including agitating by hand [29] or with a stirrer [48,49], mixing by a glass rod [51], or using a shaker with different rotating speeds. Additionally, there are diverse approaches to injecting ozone gas into water, including bubbling using a sparger [48,56] or sparging through a perforated tube [55]. It is important to note that our categorization of the ozonation methods can be quantified by parameters such as the level of agitation and the bubble size during ozone gas bubbling, which could provide more precise insights into their effects on antimicrobial effectiveness. These physical treatments can contribute to the mass transfer of ozone towards targeted microorganisms and the wettability of the surface of fresh produce and improve the reaction of ozone in water with microorganisms on fresh produce. For example, our sparging method model results indicate that the inactivation of bacteria in fresh produce with rough surfaces and complex geometries is difficult. This difficulty implies that the process may not have a high level of turbulence, although it is governed by ozone transfer. Detailed information on ozonation methods will help explain the antimicrobial action of ozone.

The influence of agricultural debris or soil on removing microorganisms from the surface of fresh produce has not been investigated. The presence of organic loads in the washing system hinders the antimicrobial effectiveness of ozonated water because ozone can oxidize organic compounds and decompose fast [83]. Further research exploring these factors, especially under various process conditions, is crucial for a comprehensive understanding of the antimicrobial effectiveness of ozonated water in fresh produce washing.

## 4. Conclusions

We have systematically evaluated the antimicrobial effectiveness of ozonated water on fresh produce washing by identifying 25 primary studies. We conducted a meta-analysis based on three ozonation methods, showing that sparging methods have a more significant impact on microbial log reductions than others, such as stationary pre-ozonated water and agitated pre-ozonated water. To explore the potential variations in the significance of process conditions influencing the antimicrobial effectiveness of ozonated water for fresh produce washing, we developed three meta-regression models using different ozonation methods since we hypothesized that different ozonation methods might yield different outcomes. These models suggest that the process conditions are critical to improving the antimicrobial effectiveness of ozonated water in different ozonation methods. The stationary pre-ozonated water method is affected by the concentration, temperature, fresh produce types, and inoculation method. The agitated pre-ozonated water method is affected by the treatment time, fresh produce types, and inoculation method. The antimicrobial effectiveness of ozonated water using sparging depends on the treatment time, fresh produce types, and inoculation method.

To the best of our knowledge, this study is the first to identify key variables and summarize the effect of all variables combined on the antimicrobial effectiveness of ozonated water on fresh produce washing. By evaluating and comparing interlaboratory studies, we have provided a comprehensive understanding of the factors influencing the effectiveness of ozonated water across different experimental settings. It provides not only important process conditions contributing to the antimicrobial effectiveness of ozonated water for fresh produce washing but also further research possibilities that have yet to be explored. The meta-regression models developed in this study will be valuable to further develop the washing process by adjusting the process conditions to enhance the antimicrobial effectiveness of ozonated water. Future research should focus on continuous washing systems, which are commonly used in industrial flume washing processes to maintain a constant dissolved ozone concentration. Although continuous ozonation systems are significant in industrial applications, they have been infrequently studied in laboratory environments. Investigating continuous washing systems in laboratory settings could bridge the gap between laboratory results and commercial applications, potentially enhancing fresh produce washing processes on a large scale.

## Figures and Tables

**Table 1:** Summary of primary studies included in the meta-analysis and study characteristics.

Ozonation Method	First Author (# of Observations)	Year	Ozone Concentration (mg/L)	Time (min)	Temperature (°C)	Inoculated Microorganisms	Fresh Produce Type	Inoculation Methods	Reference
Stationary Pre-ozonated water	A Machuca (2)	2022	0.5, 1	3	7	*E. coli* O157:H7 (surrogate)	Red chard	Spot	[39]
	H Ölmez (1)	2009	1.5	2	4, 10	*E. coli* O157:H7 (surrogate)	Lettuce	Dipping and incubation	[40]
	HG Yuk (24)	2006	1, 3, 5	0.5, 1, 3, 5	22	*E. coli* O157:H7, *L. monocytogenes*	Lettuce	Dipping	[31]
	M Nie (1)	2020	2	2	25	*E. coli* O157:H7	Bok Choy	Dipping and incubation	[41]
	RR Sharma (6)	2003	21	2, 4, 8, 16, 32, 64	4	*E. coli* O157:H7	Alfalfa sprouts	Dipping	[32]
	SME Rahman (2)	2010	5.2	3	23	*E. coli* O157:H7, *L. monocytogenes*	Spinach	Spot	[42]
	SY Tan (1)	2015	2.4	5	Room temperature	*Salmonella* spp.	Turnip	Spot	[43]
	T Ding (3)	2011	5	3	22	*E. coli* O157:H7, *Salmonella* spp., *L. monocytogenes*	Oyster mushroom	Spot	[44]
Agitated pre-ozonated water	IY Sengun (18)	2013	0.5, 1, 1.5	3, 5, 10	15	*Salmonella* spp.	Lettuce, Parsley	Dipping and incubation	[45]
	IY Sengun (9)	2014	0.5, 1, 1.5	3, 5, 10	Room temperature	*Salmonella* spp.	Carrots	Dipping and incubation	[29]
	IY Sengun (6)	2018	2	5, 10, 15	4, 15	*Salmonella* spp.	Lettuce	Spot	[46]
	LK Phua (3)	2014	2	3	25	*E. coli* O157:H7, *Salmonella* spp., *L. monocytogenes*	Mung bean sprout	Dipping	[47]
	MA And (6)	2001	23	1, 3, 5	23	*E. coli* O157:H7	Apple surface, Apple stem–calyx	Dipping and incubation	[48]
	N Singh (a) (3)	2002	10	10	22	*E. coli* O157:H7	Lettuce	Dipping	[49]
	N Singh (b) (24)	2002	5.2, 9.7, 16.5	1, 5, 10, 15	22	*E. coli* O157:H7	Lettuce, Baby carrots	Dipping	[33]
	P Pangloli (3)	2013	1.5	1, 3, 5	22	*E. coli* O157:H7	Blueberries	Spot	[50]
	Y Inatsu (8)	2011	4.64, 5.44	3	25	*E. coli* O157:H7	Lettuce, Cabbage, Spinach	Dipping	[51]
	Z Mohammad (6)	2019	5	10, 15, 20	5	*E. coli* O157:H7, *Salmonella* spp.	Alfalfa sprouts	Dipping	[52]
	Z Mohammad (2)	2020	5	15, 20	5	*E. coli* O157:H7	Alfalfa sprouts	Dipping	[53]
Sparging	A Phaephiphat (2)	2018	1	7	30	*E. coli* O157:H7 (surrogate) *Salmonella* spp.	Basil	Dipping	[54]
	GD Kumar (3)	2019	0.17, 0.18, 0.23	60, 90, 120	4	*Salmonella* spp.	Lettuce	Dipping	[55]
	H Karaca (1)	2020	12	5	5	*L. innocua*	Parsley	Dipping	[56]
	H Ölmez (9)	2010	1.5	2, 5	4, 20	*E. coli* O157:H7 (surrogate)	Lettuce	Dipping and incubation	[57]
	KL Bialka (26)	2007	1.7, 1.8, 3.7, 7.6, 7.9, 8.9, 21	2, 4, 8, 16, 32, 64	4, 20	*E. coli* O157:H7, *Salmonella* spp.	Raspberries, Strawberries	Spot	[58]
	KL Bialka (12)	2007	1.7, 1.8, 3.7, 7.6, 7.9, 8.9	2, 4, 8, 16, 32, 64	20	*E. coli* O157:H7, *Salmonella* spp.	Blueberries	Spot	[59]
	MA And (12)	2001	18, 20.8, 22, 24.5, 27.7, 36	1, 3, 5	4, 22, 23, 45	*E. coli* O157:H7	Apple surface, Apple stem–calyx	Dipping and incubation	[48]
	RR Sharma (6)	2003	21	2, 4, 8, 16, 32, 64	4	*E. coli* O157:H7	Alfalfa sprouts	Dipping	[32]
	W Xu (18)	2014	0.15, 0.25, 0.3, 0.55	1, 5, 10	4, 22, 50	*Salmonella* spp.	Grape tomato, Green onion, Lettuce	Spot	[30]

**Table 2:** Moderators used in the meta-regressions.

Variable	Values Taken ^1^	Baseline Used in Meta-Regression ^2^
Concentration	0.15 to 36 mg/L	0 mg/L
Time	0.5 to 120 min	0 min
Temperature	Low: below 15 °C Middle: above 15 °C and below 25 °C High: above 25 °C	Low
Inoculated microorganisms	*E. coli* O157:H7 (including *E. coli* O157:H7 surrogate), *Salmonella* spp., *L. monocytogenes* (including *L. innocua*)	*E. coli* O157:H7
Fresh produce types	Green leaves: Cabbage, Green onion, Lettuce, Parsley, Spinach, Red chard, Basil, Bok choy	Green leaves
	Rough surfaces and complex geometry: Alfalfa sprouts, Apple stem–calyx, Carrots, Mung bean sprouts, Oyster mushroom, Raspberries, Strawberries, Turnip	
	Smooth surfaces: Apple surface, Blueberries, Cherry tomato (or grape tomato)	
Inoculation method	Dipping, Dipping and incubation, Spot	Dipping

^1^ Values taken represents the range of continuous variables and categorical reported in primary studies; ^2^ The meta-regression model used baselines for any continuous variables and categorical variables as a default to calculate intercepts.

**Table 3:** Results of the full-moderator meta-regression by three ozonation methods predicting the microbial log reduction (log CFU/g).

Ozonation Methods			Stationary Pre-Ozonated Water		Agitated Pre-Ozonated Water		Sparging	
Parameters			Slope	*p* Value	Slope	*p* Value	Slope	*p* Value
Predictors of mean log reduction (MLR)								
Intercept		β_0_	−0.096	NS	0.573	NS	1.094	<0.05
Concentration		β_1_n__	0.063	<0.05	−0.002	NS	0.024	NS
Treatment time		β_2_n__	0.002	NS	0.071	<0.05	0.024	<0.05
Temperature	Middle	β_3_n__	0.599	<0.05	0.111	NS	0.192	NS
	High	β_3_n__	NT	NT	NT	NT	0.717	NS
Microorganism	*Salmonella* spp.	β_4_n__	−0.153	NS	0.359	NS	−0.303	NS
	*L. monocytogenes*	β_4_n__	0.003	NS	−0.375	NS	0.057	NS
Fresh produce types	Rough surface and complex geometry	β_5_n__	−0.484	<0.05	−0.108	NS	−0.957	<0.05
	Smooth surface	β_5_n__	NT	NT	1.281	<0.05	0.320	NS
Inoculation method	Dipping and Incubation	β_6_n__	0.640	<0.05	0.105	NS	0.507	NS
	Spot	β_6_n__	0.538	<0.05	0.642	<0.05	1.277	<0.05
τ^2^			0.023		0.259		0.866	
s^2^			0.020		0.261		0.899	
I^2^			72.33%		99.24%		98.95%	

NS—Moderator/variable was not statistically significant (*p* > 0.05); NT—Moderator/variable was not included in regression analysis because of insufficient data or data not being reported in primary studies.

## Data Availability

The raw data supporting the conclusions of this article will be made available by the authors on request.
